# Effect of cinnamon water extract on monocyte-to-macrophage differentiation and scavenger receptor activity

**DOI:** 10.1186/1472-6882-14-90

**Published:** 2014-03-07

**Authors:** Hee Kang, Sung-Hyun Park, Jeong-Moon Yun, Tae-Gyu Nam, Young-Eun Kim, Dae-Ok Kim, Youn Jung Kim

**Affiliations:** 1 Department of East-west Medical Science, Graduate School of East-west Medical Science, KyungHee University, Yongin, South Korea; 2 Department of Food Science and Biotechnology, College of Life Sciences, KyungHee University, Yongin, South Korea; 3 College of Nursing Science, KyungHee University, Seoul, South Korea

**Keywords:** Cinnamon water extract, Monocytes, Macrophages, Scavenger receptor, Atherosclerosis

## Abstract

**Background:**

Water soluble cinnamon extract has been shown to increase insulin sensitivity and modulate macrophage activation, a desirable trait for the management of obesity or atherosclerosis. Our present study investigated whether cinnamon water extract (CWE) may influence the differentiation of monocytes into macrophages and the activity of macrophage scavenger receptors, commonly observed in atherosclerotic lesions.

**Methods:**

We investigated the effect of CWE on the expression of various surface markers and the uptake of acetylated low density lipoprotein (LDL) in phorbol-12-myristate-13-acetate (PMA)-stimulated THP-1 cells. The protein levels of PMA or macrophage-colony stimulating factor (M-CSF)-stimulated type 1 macrophage scavenger receptor (SRA) were analyzed. Finally, the role of extracellar signal-related kinase (ERK) 1/2 in SRA synthesis and the effect of CWE on PMA-stimulated ERK1/2 were determined.

**Results:**

CWE inhibited the differentiation of monocyte by decreasing the expression of CD11b, CD36 and SRA and the uptake of acetyl LDL. CWE suppressed the upregulation of SRA by M-CSF and modulated ERK1/2 activity, which was required for PMA-induced SRA synthesis.

**Conclusions:**

Our results demonstrate that CWE was able to interfere with monocyte differentiation and macrophage scavenger activity, indicating its potential in preventing the development of atherosclerotic lesions.

## Background

Cinnamon is a popular spice around the world and is one of the oldest medicinal herbs. There is increasing evidence showing that cinnamon has anti-inflammatory, anti-viral, immunomodulating, anti-diabetic, and anti-angiogenic effects [1-8].

Circulating monocytes migrate to tissue and differentiate into macrophages [9]. Upon inflammatory stimuli, the number of recruited monocytes is increasing and they upregulate adhesion molecules and scavenger receptors required to be mature macrophages [10,11]. Although the maintenance of fully working macrophages in tissue is necessary, prolonged recruitment of monocytes into the subendothelial space where lipid metabolites can easily accumulate should be tightly controlled.

Macrophages express scavenger receptors that play an important role in host defense and tissue homeostasis [9]. Macrophages not only eradicate microbes, but also clear dying cells, remove cellular debris, and eliminate metabolite buildup in the extracellular matrix. Scavenger receptors bind and internalize exogenous and endogenous damage-associated molecules such as microbial lipopolysaccharide and lipoteichoic acid, anionic phospholipids, beta amyloid peptide, modified low density lipoprotein (LDL), and advanced glycation end products [12]. In the atherosclerotic lesion where reactive oxygen species (ROS) and macrophage-colony stimulating factor (M-CSF) are abundant, macrophages are stimulated to upregulate scavenger receptors and take up modified LDL [13,14]. Clearance of lipids through scavenger receptors is protective in early atherosclerotic lesions, but prolonged exposure to lipids causes cell death, leading to plaque instability and thrombus formation.

Among the many pharmacological effects of cinnamon described above, its insulin-enhancing activity existed only in the water soluble fraction of cinnamon [5]. Previously, we demonstrated that the water soluble cinnamon was able to suppress macrophage-derived proinflammatory cytokines in vivo [1]. In the present study, we investigate whether cinnamon water extract (CWE) may influence the differentiation of monocytes into macrophages and the activity and expression of macrophage scavenger receptors.

## Methods

### Preparation of CWE

Cinnamon bark (*Cinnamonum cassia* Presl) of Chinese origin was purchased from Omni Herb (Daegu, South Korea), and its identification was authenticated by Prof. Choi HY, Department of Herbology, KyungHee University. A voucher specimen (CC-2012) was deposited at the Laboratory of Herbology, KyungHee Unviersity. One hundred grams of cinnamon was pulverized and soaked in one liter of water for 48 h at room temperature and further dissolved by sonication for 1 h. After filtration, the resultant filtrate was evaporated and lyophilized.

### HPLC

Samples were analyzed by a reversed-phase HPLC system (Shimadzu, Kyoto, Japan) which consisted of an autosampler (SIL-20A), a binary pump (LC-20 AD), and a photodiode array detector (SPD-20A), and was equipped with a Zorbax Eclipse XDB-C18 column (5 μm × 4.6 mm × 250 mm) (Agilent Technologies, CA, USA). Gradient flows for the two-solvent system (solvent A, 0.1% formic acid in water; solvent B, 0.1% formic acid in acetonitrile) were as follows: 95% A/5% B at 0 min, 85% A/15% B at 10 min, 70% A/30% B at 40 min, 50% A/50% B at 70 min, 40% A/60% B at 75 min, 30% A/70% B at 80 min, 95% A/5% at 85 min, and 95% A/5% at 90 min. The flow rate of the mobile phase was 0.6 ml/min with an injection volume of 20 μl. Detection was at 254 nm for catechin, epicatechin, coniferyl aldehyde, coumarin, cinnamic acid, and cinnam aldehyde (Sigma, MO, USA), or at 280 nm for cinnamyl alcohol (Sigma). Compounds from CWE were tentatively identified with a spiked input of authentic standards, in addition to the comparison of their retention time and UV-visible spectral patterns.

### Mice

Male Balb/c mice aged 8–10 weeks were purchased from the Korean branch of Taconic, SamTaco (Osan, Korea), and maintained with rodent chow and water ad libitum in a temperature and humidity controlled pathogen-free facility at KyungHee University. Mice were cared for according to the guidelines issued by the Guide for the Care and Use of Laboratory Animals issued by the United States National Research Council (1996), and the protocol was approved by the KyungHee University institutional committee for the care and use of laboratory animals (KHUSASP(GC)-10-001).

### THP-1 culture

THP-1 cells were obtained from the Korean Cell Line Bank (Seoul, South Korea). Cells were maintained in RPMI 1640 medium (Hyclone, Utah, USA) supplemented with 25 mM HEPES, 1% penicillin-streptomycin, and 10% fetal bovine serum (FBS) (Hyclone) at 37°C under 5% CO_2_.

### Peritoneal macrophage culture

Peritoneal macrophages were isolated from mice three days after intraperitoneal injection of 2 ml of 4% thioglycollate (BD, Sparks, MD, USA). Cells were cultured overnight in DMEM supplemented with 1% penicillin-streptomycin and 10% FBS. After incubation overnight, nonadherent cells were removed.

### MTT assay

The MTT method was used to examine the number of phorbol-12-myristate-13-acetate (PMA)-stimulated cells. THP-1 cells (4 × 10^4^ cells / 0.1 ml) were plated in 96-well plates and treated with 10, 50, and 100 μg/ml CWE in the presence of 100 nM PMA for 24, 48 and 72 h. After 2 h of incubation with 10 μl MTT solution (5 mg/ml) (AMRESCO, Solon, Ohio, USA), the medium was aspirated, and 100 μl dimethyl sulfoxide was added. The optical density was read at 560 nm using a microplate reader (Molecular Devices, Sunnyvale, CA, USA).

### cDNA preparation and real-time PCR

THP-1 cells (2 × 10^6^ cells) were cultured with PMA (100 nM) in the presence of CWE for 48 h. Total RNA was extracted using an RNeasy mini kit (Qiagen, Germany) according to the manufacturer’s instructions, and revere transcribed using Superscript III reverse transcriptase (Invitrogen, CA, USA). The cDNA obtained was mixed with Power SYBR Green PCR Master mix (Applied Biosystems, CA, USA) and 2 pmol primers in a final volume of 20 μl. The following forward and reverse primer sequences were used: SRA, forward: 5′- GCA GTG GGA TCA CTT TCA CAA- 3′ and reverse: 5′AGC TGT CAT TGA GCG AGC ATC-3′; CD36, forward: 5′- GCC AAG GAA AAT GTA ACC CAG G −3′ and reverse: 5′- GCC TCT GTT CCA ACT GAT AGT GA-3′; and actin, forward: 5′-GCAAATGCTTCTAGGCGGACTAT-3′ and reverse: 5′- TGTTTTCTGCGCAAGTTAGGTTT- 3′. PCR was performed in triplicate using a StepOne Real-time PCR system (Applied Biosystems). After an initial heat denaturation at 95°C for 10 min, the PCR conditions were set for 40 cycles at 95°C for 15 s and 60°C for 1 min. Relative gene expression was determined using the standard curve method and normalized to actin.

### Western blotting

THP-1 cells (1 × 10^7^ cells) were pretreated with CWE or U0126 (20 μM) for 1 h prior to exposure to PMA for 30 min for ERK1/2 activity or for 72 h for SRA synthesis. Mouse macrophages (5 × 10^6^ cells) were cultured with M-CSF (25 ng/ml) (R&D Systems, MN, USA) in the presence of CWE for 24 h. Cells were rinsed in cold PBS and then lysed on ice in RIPA buffer (50 mM Tris–HCl, pH 7.5; 150 mM NaCl; 1 mM EDTA; 20 mM NaF; 0.5% NP-40; and 1% Triton X-100) containing phosphatase inhibitor cocktail (Sigma) and protease inhibitor cocktail (Roche Diagnostics, Germany). After centrifugation at 13,000 × g for 10 min, supernatants were collect, and protein concentrations were determined using the Bradford protein assay reagent (Bio-Rad, USA). The samples were separated on an 8% or 10% SDS-polyacrylamide gel and were transferred to polyvinylidene fluoride membranes. The membranes were blocked with 5% skim milk in Tris-buffered saline with 0.1% Tween 20 (TBST) for 1 h. The membranes were incubated with antibodies against human SRA, actin, GAPDH (Santa Cruz Biotechnology, CA, USA), mouse SRA (R&D Systems), extracellular signal-related kinase (ERK) 1/2, or phospho-ERK1/2 ( Cell Signaling Technology, MA, USA ) diluted in 5% skim milk in TBST overnight at 4°C. The blots were washed with TBST and incubated for 1 h with anti-goat, mouse or rabbit horseradish peroxidase-conjugated antibodies. Immunoreactive bands were visualized by enhanced chemiluminescence using BioFx (SurModics, MN, USA).

### Flow cytometry

Cells were incubated with Alexa^488^-acetyl LDL (2.5 μg/ml) (Molecular Probes, USA) for 16 h in PMA-stimulated THP-1 cells or for 2 h in mouse peritoneal macrophages. Fucoidan (75 μg/ml) (Sigma) or U0126 (20 μM) (Sigma) was incubated 24 h before the addition of Alexa^488^-acetyl LDL. Cells were washed and harvested with PBS and then analyzed on a FACSAria (BD Biosciences) with the use of FACSDiva software. For every sample, the mean fluorescence intensity of cells was determined.

### Statistical analysis

Statistical analysis was performed using student *t* test or one-way analysis of variances followed by Dunnet’s test for multiple comparison. Calculations were carried out using the SPSS version 20. *P* values less than 0.05 were considered significant.

## Results

### HPLC analysis

**Figure 1:**
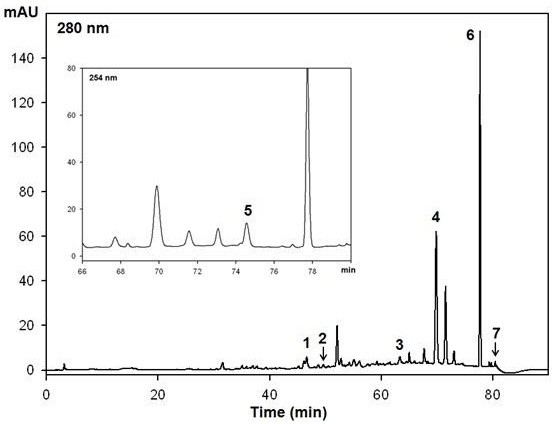
**The HPLC chromatogram of cinnamon water extract (CWE).** Peak identification: 1, catechin; 2, epicatechin; 3, coniferyl aldehyde; 4, coumarin; 5, cinnamyl alcohol; 6, cinnamic acid; 7, cinnamaldehyde.

We identified the known bioactive compounds of cinnamon, cinnamyl alcohol, cinnamic acid, cinnamaldehyde, coniferyl aldehyde, and coumarin (Figure 1). In addition, we confirmed the presence of the water soluble polyphenols, catechin and epicatechin in CWE.

### Effect of CWE on differentiation marker expression

**Figure 2:**
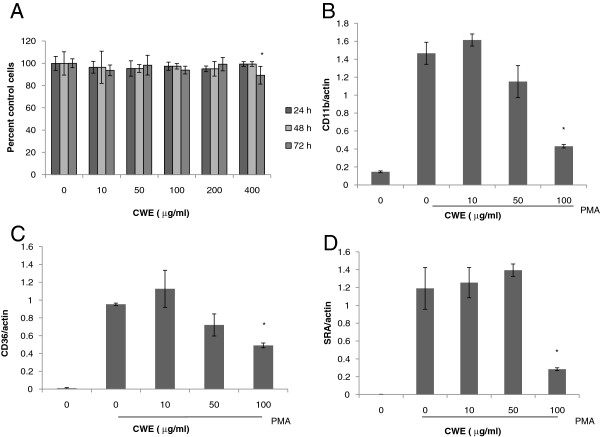
**Effect of CWE on the expression of differentiation markers. A**: THP-1 cells were incubated with different concentrations of CWE in the presence of PMA (100 nM) for 24, 48 and 72 h. Cell viability was measured by MTT assay. **B-D**: THP-1 cells were incubated with CWE in the presence of PMA for 48 h. Total RNA was extracted and mRNA expression was analyzed using real-time PCR. Each gene was normalized with actin. Data are presented as mean ± SD of three independent experiments. * *p* <0.05, indicating significant difference from cells treated with PMA only.

The human monocytic cell line THP-1 is a suspension cell type resembling circulating primary monocytes. Treatment with PMA causes these cells to stop mitosis, adhere to the culture plate, and differentiate into mature macrophages [15]. Before assessing the bioactivity of CWE, we examined whether CWE caused cytotoxicity in PMA-stimulated cells. CWE was not toxic to cells for 24 and 48 h, but the maximum concentration assayed here (400 μg/ml) caused a 10% decrease in cell viability at the end of 72 h culture period (Figure 2A). Then, we measured whether CWE affects gene expression of the scavenger receptors, type 1 macrophage scavenger receptor (SRA) and CD36, and the macrophage-specific integrin, CD11b using real-time PCR. These differentiation markers are upregulated in response to PMA [16-18]. CWE treatment reduced the expression levels of CD11b and CD36 in a concentration-dependent manner (Figures 2B,C). The reduction of SRA gene occurred only at 100 μg/ml, but the magnitude was marked compared with the other differentiation markers (Figure 2D).

### Effect of CWE on scavenger receptor activity in differentiating human monocytes and mouse macrophages

**Figure 3:**
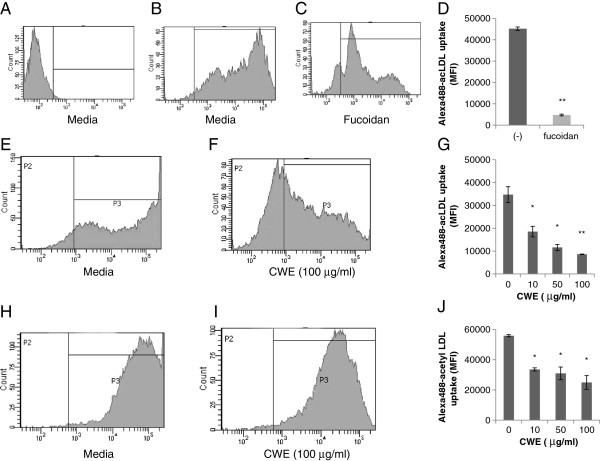
**Effect of CWE on scavenger receptor activity. A-D**: Mouse peritoneal macrophages were cultured with or without fucoidan (75 μg/ml) for 24 h and then incubated with Alexa^488^-acetyl LDL for 2 h. Cellular uptake of Alexa^488^-acetyl LDL was analyzed by flow cytometry. **A**, cells without Alexa^488^-acetyl LDL; **B**, cells with Alexa^488^-acetyl LDL; **C**, cells with Alexa^488^-acetyl LDL in the presence of fucoidan. **E-G**: THP-1 cells were stimulated with PMA (100 nM) for 24 h in the presence of CWE and then incubated with Alexa^488^-acetyl LDL for 16 h. **E**, cells treated with PMA only. **F**, cells treated with PMA plus CWE (100 μg/ml). **H-J**: Mouse peritoneal macrophages were cultured with CWE for 24 h and then incubated with Alexa^488^-acetyl LDL for 2 h. **D, G, J**: Results are presented as mean fluorescence intensity (MFI) ± SD of three independent assays. * *p* <0.05, ** *p* < 0.01 indicating significant difference from cells without treatment.

SRA is the main scavenger receptors for the uptake of modified LDL [19]. Fucoidan, polysaccharide sulfate ester isolated from brown seaweed, competes with SRA for acetyl LDL [20]. Mouse peritoneal macrophages were cultured in the presence of fucoidan for 24 h and then incubated with Alexa^488^-acetyl LDL for 2 h. In the case of peritoneal macrophages, incubation of longer than 2 h did not increase the uptake of Alexa^488^-acetyl LDL (data not shown). In our experimental setting, fucoidan inhibited the uptake of Alexa^488^-acetyl LDL by 80% (Figures 3A-D).

Since an elevated uptake of modified LDL is characteristic of monocyte differentiation [17], we measured whether CWE may influence the activity of scavenger receptors in differentiating THP-1 cells. Cells were stimulated for 24 h with PMA in the presence of CWE and then incubated with Alexa^488^-acetyl LDL for 16 h. CWE suppressed Alexa^488^-acetyl LDL uptake in a concentration-dependent manner by 47%, 67% and 75% at 10, 50 and 100 μg/ml CWE, respectively (Figures 3E-G). CWE also exerted a similar inhibition in primary macrophage culture: Alexa^488^-acLDL uptake was reduced by 40%, 45% and 55% at 10, 50 and 100 μg/ml CWE, respectively (Figures 3H-J).

### Effect of CWE on M-CSF-induced SRA protein synthesis

**Figure 4:**
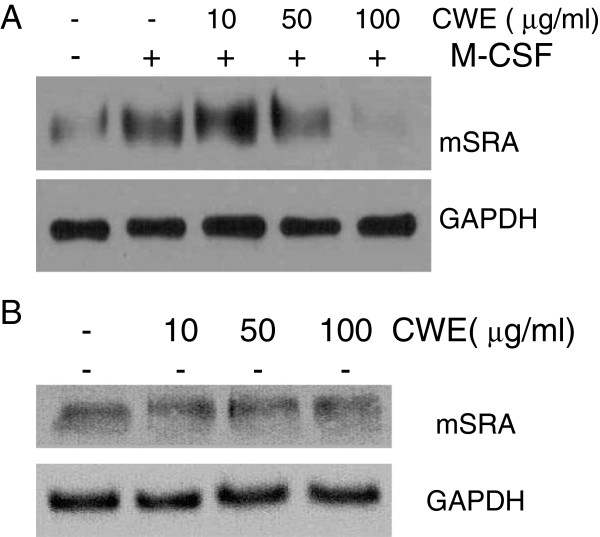
**Effect of CWE on SRA synthesis.** Peritoneal macrophages were cultured in the presence of CWE with **(A)** or without **(B)** M-CSF for 24 h, and whole protein was extracted. SRA protein was analyzed by Western blotting. GAPDH was used as endogenous control. One representative Western blot of three or four experiments is shown.

In order to make mouse peritoneal macrophages upregulate SRA expression, we stimulated the cells with M-CSF, a cytokine that promotes the differentiation of monocytes and enhances SRA expression in macrophages [20-22]. M-CSF treatment enhanced SRA levels compared with non-treated cells, and CWE at 100 μg/ml almost abolished SRA protein synthesis (Figure 4A). CWE alone appeared not to affect the constitutive synthesis of SRA protein (Figure 4B).

### Effect of CWE on PMA-induced ERK1/2 activity

**Figure 5:**
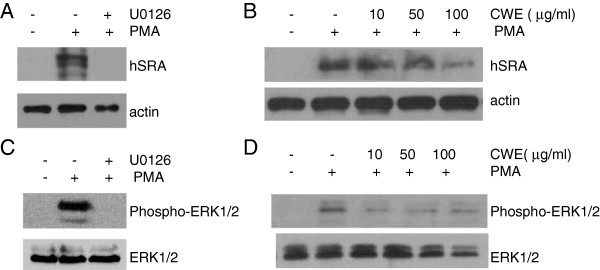
**Effect of CWE on PMA-stimulated ERK1/2 activation. A-B**: THP-1 cells were incubated with or without U0126 (20 μM) or CWE in the presence of PMA for 72 h. **C-D**: THP-1 cells were preincubated with or without U0126 (20 μM) or CWE for 1 h and then stimulated with PMA for 30 min. SRA protein and phospho-ERK1/2 were analyzed by Western blotting. Actin or ERK1/2 was used as endogenous control. One representative Western blot of four experiments is shown.

PMA stimulates THP-1 cells to enter cell arrest via a Raf-MEK-ERK1/2 signaling pathway, after which they become differentiated [16,23]. We tried to identify the role of ERK1/2 in PMA-induced SRA synthesis. Treatment with U0126, an inhibitor of ERK1/2 activation, strongly inhibited SRA protein in PMA-stimulated THP-1 cells (Figure 5A). We found that U0126 completely inhibited the attachment of cells into the culture plate and thus blocked the uptake of acetyl LDL (data not shown). SRA protein synthesis in PMA-differentiated THP-1 cells was suppressed at 100 μg/ml of CWE (Figure 5B). CWE also inhibited PMA-stimulated ERK1/2 activation but in a concentration- independent manner (Figure 5D).

## Discussion

Since macrophages respond to modified LDL, fatty acids, and other lipid metabolites, persistent low grade inflammation is observed in metabolic disorders such as obesity, type II diabetes and atherosclerosis [24,25]. LDL oxidation in the vascular wall triggers the activation of resident macrophages, recruitment and differentiation of blood monocytes, and the formation of lipid-laden foam cells [13]. LDL could be oxidized by various stimuli but any chronic inflammatory environment further promotes LDL oxidation. Thus traditional herbal medicines that have been proved to be anti-inflammatory can be a good resource to prevent or ameliorate atherosclerosis.

Since treatment of THP-1 cells with PMA induces differentiation into macrophages, this cell line is widely used for atherosclerotic research [15]. CD11b is induced not only in PMA-stimulated THP-1 cells but also in oxidized LDL-stimulated mouse monocytes in vivo [18]. It forms a noncovalently linked heterodimer with the common β-subunit CD18 and binds to various adhesion molecules in the endothelial cells and extracellular matrix, therefore mediating the migration and adhesion of cells during monocyte differentiation [11,26]. Our result indicates that CWE can reduce CD11b dependent monocyte recruitment in the atherogenic lesion.

Scavenger receptors consist of eight classes; among them, SRA, belonging to class A, is expressed mostly in macrophages [12,27]. CD36, one of the class B scavenger receptors, is distributed in a variety of cells including platelets, cells with hematopoietic origin, adipocytes, and endothelial cells [28]. The expression of SRA and CD36 can be differentially controlled. Peroxisome-proliferator-activated γ, a transcription factor that is involved in lipid metabolism and monocyte differentiation, influences the expression of CD36, but not of SRA [29]. The different response of SRA and CD36 genes to CWE treatment indicate that CWE may separately affect PMA-activated signaling pathways that express these genes. It should be noted here that inhibition of acetyl LDL uptake in THP-1 cells by CWE was more marked than that of SRA and CD36 expression. A similar effect was observed in peritoneal macrophages of which scavenger receptors had already occupied before CWE treatment. We postulate that since CWE is likely to have high molecular weight polysaccharides, it may have some activity that competitively binds with scavenger receptors, as does fucoidan.

M-CSF is produced by a variety of cells including monocytes, macrophages, and endothelial cells, and its production increases in atherosclerotic lesions [21,22,30,31]. Our results showed that CWE acted on the M-CSF-induced SRA synthesis pathway, but not on its endogenous production. These results suggest that CWE may contribute to the prevention of foam cell development that results from the excessive uptake of lipid metabolites by SRA.

PMA, an analogue of diacylglycerol, activates protein kinase C (PKC), which is a key enzyme of cell differentiation. PMA treatment causes cell cycle arrest in THP-1 cells through the upregulation of p21, a negative regulator of cyclin dependent kinases [32]; the increased expression of p21 is due to the generation of ROS and PKC-mediated ERK1/2 activation. ROS inhibitors are reported to inhibit the level of SRA gene expression and acetyl LDL uptake in PMA-stimulated THP-1 cells [17]. This may explain why some antioxidants reduce atherosclerosis [33]. Cinnamon has antioxidant hydrophilic polyphenols such as catechin, epicatechin, and tannins [6,34]. Our previous study showed that the high molecular fraction of CWE (over 10 kDa) occupied most of its phenolics and was responsible for its inhibitory effect on LPS-induced ERK1/2 and other inflammatory signaling molecules [1]. The same components in CWE may exert a similar effect on PMA-mediated ERK1/2 activity. However, the inhibition of SRA and other surface molecules by CWE cannot be fully accounted for by its effect on ERK1/2 because effective inhibitory concentration ranges are different. Presumably, lower concentrations of CWE have more of unidentified stimulatory ingredients that affect the pathways linking ERK1/2 to SRA synthesis.

## Conclusions

Taken together, CWE was able to interfere with monocyte differentiation and macrophage scavenger activity, indicating its potential in preventing the development of atherosclerotic lesions. Further studies are required to validate the protective effect of CWE using in vivo models.

## Abbreviations

CWE: Cinnamon water extract; SRA: Type 1 macrophage scavenger receptor; LDL: Low density lipoprotein; ERK 1/2: Extracellular signal-related kinase 1/2; M-CSF: Macrophage-colony stimulating factor; ROS: Reactive oxygen species; PKC: Protein kinase C.

## Competing interests

The authors declare that they have no conflict of interest.

## Authors’ contributions

HK designed research, performed realtime PCR and wrote the paper. JMY performed the western blot assay. YEK performed the MTT assay. SHB, TGN, and DOK performed HPLC. YJK participated in the study design. All authors read and approved the final manuscript.

## Pre-publication history

The pre-publication history for this paper can be accessed here:

http://www.biomedcentral.com/1472-6882/14/90/prepub

## References

[B1] Hong JW Yang GE Kim YB Eom SH Lew JH Kang H Anti-inflammatory activity of cinnamon water extract in vivo and in vitro LPS-induced models BMC Complement Altern Med 2012 12 237 10.1186/1472-6882-12-237 23190501 PMC3533872

[B2] Liao JC Deng JS Chiu CS Hou WC Huang SS Shie PH Huang GJ Anti-inflammatory activities of cinnamomum cassia constituents in vitro and in vivo Evid Based Complement Alternat Med 2012 2012 429320 22536283 10.1155/2012/429320 PMC3318905

[B3] Reddy AM Seo JH Ryu SY Kim YS Kim YS Min KR Kim Y Cinnamaldehyde and 2-methoxycinnamaldehyde as NF-kappaB inhibitors from Cinnamomum cassia Planta Med 2004 70 9 823 827 10.1055/s-2004-827230 15503352

[B4] Zhuang M Jiang H Suzuki Y Li X Xiao P Tanaka T Ling H Yang B Saitoh H Zhang L Qin C Sugamua K Hattori T Procyanidins and butanol extract of Cinnamomi Cortex inhibit SARS-CoV infection Antiviral Res 2009 82 1 73 81 10.1016/j.antiviral.2009.02.001 19428598 PMC7114128

[B5] Lee BJ Kim YJ Cho DH Sohn NW Kang H Immunomodulatory effect of water extract of cinnamon on anti-CD3-induced cytokine responses and p38, JNK, ERK1/2, and STAT4 activation Immunopharmacol Immunotoxicol 2011 33 4 714 722 10.3109/08923973.2011.564185 22053946

[B6] Anderson RA Broadhurst CL Polansky MM Schmidt WF Khan A Flanagan VP Schoene NW Graves DJ Isolation and characterization of polyphenol type-A polymers from cinnamon with insulin-like biological activity J Agric Food Chem 2004 52 1 65 70 10.1021/jf034916b 14709014

[B7] Kwon HK Jeon WK Hwang JS Lee CG So JS Park JA Ko BS Im SH Cinnamon extract suppresses tumor progression by modulating angiogenesis and the effector function of CD8+ T cells Cancer Lett 2009 278 2 174 182 10.1016/j.canlet.2009.01.015 19203831

[B8] Lu J Zhang K Nam S Anderson RA Jove R Wen W Novel angiogenesis inhibitory activity in cinnamon extract blocks VEGFR2 kinase and downstream signaling Carcinogenesis 2009 31 3 481 488 19969552 10.1093/carcin/bgp292 PMC3105590

[B9] Zhang X Mosser DM Macrophage activation by endogenous danger signals J Pathol 2008 214 2 161 178 10.1002/path.2284 18161744 PMC2724989

[B10] Geng Y Kodama T Hansson GK Differential expression of scavenger receptor isoforms during monocyte-macrophage differentiation and foam cell formation Arterioscler Thromb 1994 14 5 798 806 10.1161/01.ATV.14.5.798 8172856

[B11] Prudovsky I Popov K Akimov S Serov S Zelenin A Meinhardt G Baier P Sohn C Hass R Antisense CD11b integrin inhibits the development of a differentiated monocyte/macrophage phenotype in human leukemia cells Eur J Cell Biol 2002 81 1 36 42 10.1078/0171-9335-00219 11893077

[B12] Moore KJ Freeman MW Scavenger receptors in atherosclerosis: beyond lipid uptake Arterioscler Thromb Vasc Biol 2006 26 8 1702 1711 10.1161/01.ATV.0000229218.97976.43 16728653

[B13] Yoshida H Kisugi R Mechanisms of LDL oxidation Clin Chim Acta 2010 411 23–24 1875 1882 20816951 10.1016/j.cca.2010.08.038

[B14] Lundberg AM Hansson GK Innate immune signals in atherosclerosis Clin Immunol 2010 134 1 5 24 10.1016/j.clim.2009.07.016 19740706

[B15] Qin Z The use of THP-1 cells as a model for mimicking the function and regulation of monocytes and macrophages in the vasculature Atherosclerosis 2012 221 1 2 11 10.1016/j.atherosclerosis.2011.09.003 21978918

[B16] Schwende H Fitzke E Ambs P Dieter P Differences in the state of differentiation of THP-1 cells induced by phorbol ester and 1,25-dihydroxyvitamin D3 J Leukoc Biol 1996 59 4 555 561 8613704

[B17] De Kimpe SJ Anggard EE Carrier MJ Reactive oxygen species regulate macrophage scavenger receptor type I, but not type II, in the human monocytic cell line THP-1 Mol Pharmacol 1998 53 6 1076 1082 9614211

[B18] Fuhrman B Partoush A Volkova N Aviram M Ox-LDL induces monocyte-to-macrophage differentiation in vivo: Possible role for the macrophage colony stimulating factor receptor (M-CSF-R) Atherosclerosis 2008 196 2 598 607 10.1016/j.atherosclerosis.2007.06.026 17675037

[B19] Kunjathoor VV Febbraio M Podrez EA Moore KJ Andersson L Koehn S Rhee JS Silverstein R Hoff HF Freeman MW Scavenger receptors class A-I/II and CD36 are the principal receptors responsible for the uptake of modified low density lipoprotein leading to lipid loading in macrophages J Biol Chem 2002 277 51 49982 49988 10.1074/jbc.M209649200 12376530

[B20] Nikolic D Calderon L Du L Post SR SR-A ligand and M-CSF dynamically regulate SR-A expression and function in primary macrophages via p38 MAPK activation BMC Immunol 2011 12 37 10.1186/1471-2172-12-37 21736734 PMC3141791

[B21] de Villiers WJ Fraser IP Hughes DA Doyle AG Gordon S Macrophage-colony-stimulating factor selectively enhances macrophage scavenger receptor expression and function J Exp Med 1994 180 2 705 709 10.1084/jem.180.2.705 8046345 PMC2191609

[B22] Clinton SK Underwood R Hayes L Sherman ML Kufe DW Libby P Macrophage colony-stimulating factor gene expression in vascular cells and in experimental and human atherosclerosis Am J Pathol 1992 140 2 301 316 1739124 PMC1886415

[B23] Ueda Y Hirai S Osada S Suzuki A Mizuno K Ohno S Protein kinase C activates the MEK-ERK pathway in a manner independent of Ras and dependent on Raf J Biol Chem 1996 271 38 23512 23519 10.1074/jbc.271.38.23512 8798560

[B24] Prieur X Roszer T Ricote M Lipotoxicity in macrophages: evidence from diseases associated with the metabolic syndrome Biochim Biophys Acta 2010 1801 3 327 337 10.1016/j.bbalip.2009.09.017 19796705

[B25] Onat A Metabolic syndrome: nature, therapeutic solutions and options Expert Opin Pharmacother 2011 12 12 1887 1900 10.1517/14656566.2011.585462 21756201

[B26] Bohbot A Eischen A Felden C Vincent F Oberling F U937 cell line: impact of CSFs, IL-6 and IFN-gamma on the differentiation and the Leu-CAM proteins expression Exp Hematol 1993 21 4 564 572 8096463

[B27] Stephen SL Freestone K Dunn S Twigg MW Homer-Vanniasinkam S Walker JH Wheatcroft SB Ponnambalam S Scavenger receptors and their potential as therapeutic targets in the treatment of cardiovascular disease Int J Hypertens 2010 2010 2010 Article ID 646929 10.4061/2010/646929 PMC2958427 20981357

[B28] Collot-Teixeira S Martin J McDermott-Roe C Poston R McGregor JL CD36 and macrophages in atherosclerosis Cardiovasc Res 2007 75 3 468 477 10.1016/j.cardiores.2007.03.010 17442283

[B29] Moore KJ Rosen ED Fitzgerald ML Randow F Andersson LP Altshuler D Milstone DS Mortensen RM Spiegelman BM Freeman MW The role of PPAR-gamma in macrophage differentiation and cholesterol uptake Nat Med 2001 7 1 41 47 10.1038/83328 11135614

[B30] Rambaldi A Young DC Griffin JD Expression of the M-CSF (CSF-1) gene by human monocytes Blood 1987 69 5 1409 1413 3105621

[B31] Rajavashisth TB Andalibi A Territo MC Berliner JA Navab M Fogelman AM Lusis AJ Induction of endothelial cell expression of granulocyte and macrophage colony-stimulating factors by modified low-density lipoproteins Nature 1990 344 6263 254 257 10.1038/344254a0 1690354

[B32] Traore K Trush MA George M Jr Spannhake EW Anderson W Asseffa A Signal transduction of phorbol 12-myristate 13-acetate (PMA)-induced growth inhibition of human monocytic leukemia THP-1 cells is reactive oxygen dependent Leuk Res 2005 29 8 863 879 10.1016/j.leukres.2004.12.011 15978937

[B33] Shiao MS Chiu JJ Chang BW Wang J Jen WP Wu YJ Chen YL In search of antioxidants and anti-atherosclerotic agents from herbal medicines BioFactors 2008 34 2 147 157 10.1002/biof.5520340206 19706980

[B34] Peng X Cheng KW Ma J Chen B Ho CT Lo C Chen F Wang M Cinnamon bark proanthocyanidins as reactive carbonyl scavengers to prevent the formation of advanced glycation endproducts J Agric Food Chem 2008 56 6 1907 1911 10.1021/jf073065v 18284204

